# A cDNA microarray, UniShrimpChip, for identification of genes relevant to testicular development in the black tiger shrimp (*Penaeus monodon*)

**DOI:** 10.1186/1471-2199-12-15

**Published:** 2011-04-12

**Authors:** Rungnapa Leelatanawit, Umaporn Uawisetwathana, Sirawut Klinbunga, Nitsara Karoonuthaisiri

**Affiliations:** 1 National Center for Genetic Engineering and Biotechnology (BIOTEC), National Science and Technology Development Agency (NSTDA), Klong 1, Klong Luang, Pathumthani 12120, Thailand; 2 Center of Excellence for Marine Biotechnology, Faculty of Science, Chulalongkorn University, Bangkok 10330, Thailand

**Keywords:** shrimp, *Penaeus monodon*, cDNA microarrays, testis, *CSN5*, *CSN6*

## Abstract

**Background:**

Poor reproductive maturation in captive male broodstock of the black tiger shrimp (*Penaeus monodon*) is one of the serious problems to the farming industries. Without genome sequence, EST libraries of *P. monodon *were previously constructed to identify transcripts with important biological functions. In this study, a new version of cDNA microarray, UniShrimpChip, was constructed from the *Peneaus monodon *EST libraries of 12 tissues, containing 5,568 non-redundant cDNA clones from 10,536 unique cDNA in the *P. monodon *EST database. UniShrimpChip was used to study testicular development by comparing gene expression levels of wild brooders from the West and East coasts of Thailand and domesticated brooders with different ages (10-, 14-, 18-month-old).

**Results:**

The overall gene expression patterns from the microarray experiments revealed distinct transcriptomic patterns between the wild and domesticated groups. Moreover, differentially expressed genes from the microarray comparisons were identified, and the expression patterns of eight selected transcripts were subsequently confirmed by reverse-transcriptase quantitative PCR (RT-qPCR). Among these, expression levels of six subunits (*CSN2, 4, 5, 6, 7a*, and *8) *of the *COP9 signalosome (CSN) *gene family in wild and different ages of domesticated brooders were examined by RT-qPCR. Among the six subunits, *CSN5 *and *CSN6 *were most highly expressed in wild brooders and least expressed in the 18-month-old domesticated group; therefore, their full-length cDNA sequences were characterized.

**Conclusions:**

This study is the first report to employ cDNA microarray to study testicular development in the black tiger shrimp. We show that there are obvious differences between the wild and domesticated shrimp at the transcriptomic level. Furthermore, our study is the first to investigate the feasibility that the *CSN *gene family might have involved in reproduction and development of this economically important species.

## Background

Even though Thailand was a leading producer of cultured black tiger shrimp (*Penaeus monodon)*, generating at least 200,000 metric tons with an income of over 2 billion USD annually in the nineties [1], the shrimp industry has deteriorated due to widespread disease, slow growth rate, and low quality broodstock.

The reproductive maturation of both males and females of penaeid shrimp are poorly understood [2], and the males have been studied even less than in the females [3]. Reduced degrees of maturation are usually observed in the captive brooders compared to the wild brooders. For instance, when mated with wild males, the wild females gave higher egg production and % fertilized eggs than the pond-reared females. Similarly, when mated with wild females, the wild males gave higher % fertilized eggs and % nauplii than the pond-reared males [4]. In females, the low degrees of reproductive maturation in captivity can be relieved by unilateral eyestalk ablation technique that can induce ovarian development and ovulation [2]. However, the technique does not affect the male reproductive maturation [5]. Moreover, there has not been any report on optimal age of the males for mating though a previous study in *P. monodon *suggested that age positively correlated to the gonad weight, spermatophore weight, sperm count and % normal sperm [6]. Therefore, it is important to understand fundamental differences in molecular mechanisms between domesticated and wild-caught male broodstock in order to improve farming conditions and sustain the aquaculture of this species.

Various efforts have been made to study the fundamental mechanism of male reproduction in this species. Leelatanawit et al. constructed subtractive cDNA libraries of *P. monodon *ovaries and testes to identify gender-specific transcripts [7]. Unfortunately, almost all of the preferentially expressed ESTs in the *P. monodon *testis were unknown transcripts (96.7%). A search for genes differentially expressed in testes was continued by constructing conventional and subtractive EST libraries from testis of *P. monodon *to identify several reproduction-related genes such as *PMTST1 *(*P. monodon testis-specific transcript 1*), *spermatogonial stem-cell renewal factor, transformer 2 *(*Tra-2*), *progestin receptor membrane component 1 *(*PGRMC1*) and *Dmc1 *[8,9]. Recently, more testis ESTs (4,803 ESTs) were sequenced and analyzed to indentify more testis-relevant genes in this organism [10]. These previous studies started to shed some light onto potential genes involved in testicular development. Yet, further investigation is needed to understand this process in *P. monodon*.

Microarray technology can simultaneously monitor gene expression levels of a large number of genes [11]. Previously, we have constructed a reproduction-specific cDNA microarray (*Repro*Array^GTS^) to study different stages of the ovarian development of the black tiger shrimp and able to identify many important reproduction-related genes exhibiting a differential expression profile during its ovarian development such as *nuclear autoantigenic sperm protein *(*NASP*) [12].

In this study, we have constructed another version of microarray containing unigenes to include more genes on the array for more diverse studies of this species. This is the first report in the black tiger shrimp to use the microarray technique to study testicular development. The microarray results identified differentially expressed gene clusters which might be involved in differences between domesticated and wild-caught male broodstock. Some of these genes (i.e. different isoforms of *constitutive photomorphogenic 9 signalosome *(*CSN*)) were further characterized for their possible roles on testicular development.

## Results

### Features of UniShrimpChip

**Figure 1:**
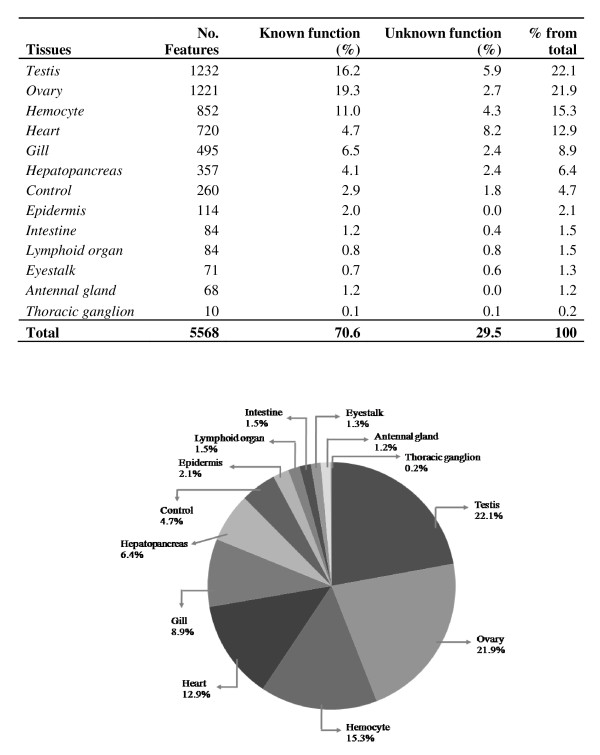
**Summary of the tissue origin for the features used on the microarray (UniShrimpChip)**.

**Figure 2:**
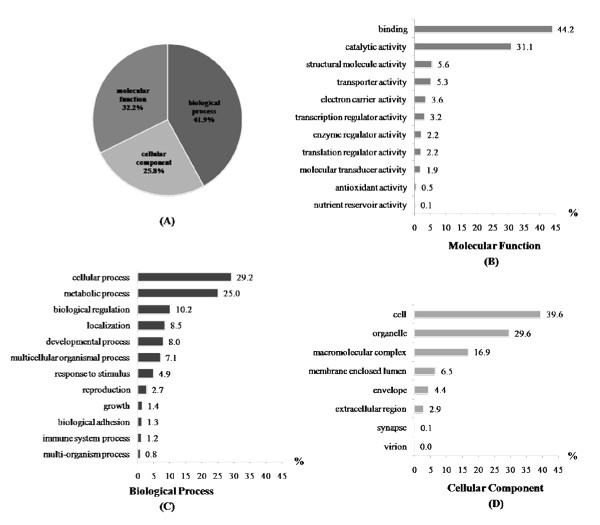
**Functional categories by Gene Ontology (GO) analysis of UniShrimpChip features**. (A) Pie diagrams show the percentage distribution of sequences among the three principal GO categories: (B) molecular functions, (C) biological processes and (D) cellular components. The percentage of sequence numbers in each category was calculated from the total sequence numbers assigned in that particular category.

A cDNA microarray, UniShrimpChip, was constructed from 5,568 sequences of the previously established 12 EST libraries [13] and spotted in duplicate on each microarray slide (Figure 1). A total of 260 control spots include both positive (shrimp genomic DNA) and negative controls (buffer, bacteria DNA, virus DNA). The highest numbers of features on UniShrimpChip was sequences found in testis and ovary EST libraries (about 22% each) of *P. monodon*. A total of ~70% of the features were homologues of genes with putative known functions (Figure 1). The features of UniShrimpChip were further categorized according to the Gene Ontology (Figure 2A). The highest numbers of features belonged to the "biological function" (42%) followed by the "molecular function" (32%) and the "cellular components" (26%).

### Large-Scale Gene Expression Analysis in Testis of Penaeus monodon

**Figure 3:**
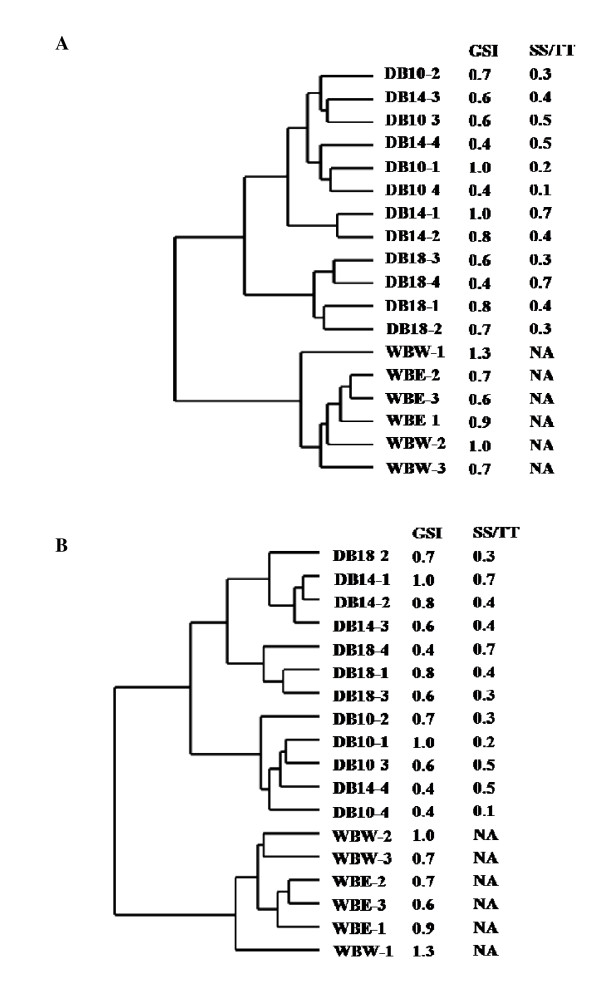
**Cluster analysis of overall gene expression patterns from wild and domesticated male broodstock (A) using all transcripts on the UniShrimpChip and (B) using only transcripts from testis ESTs**. WBW and WBE are wild broodstock from the West and East coasts of Thailand, respectively. DB10, DB14, and DB18 are 10-, 14, and 18-month-old domesticated broodstock, respectively. GSI is a gonadosomatic index value calculated from the percentage of gonad weight by total body weight. SS/TT is a ratio of sperm sac weight to testis weight.

UniShrimpChip was used to compare gene expression profiles of testes from wild (West and East coasts of Thailand) and domesticated brooders (Cy5, Red) by using pooled testes from juveniles as a common references (Cy3, Green) (Table 1). From the total of the 18 microarray comparisons, the overall expression patterns of every transcript were clustered to identify relationship among the brooder types (Figure 3). Hierarchical cluster revealed distinct differences in expression patterns between wild and domesticated brooders. Among domesticated shrimp at different ages, the 18-month-old group was clearly clustered as a separated group from the other cluster that contained both 10- and 14-month-old shrimp; Figure 3A. However, if only gene expression patterns of transcripts from the testis library (1,076 transcripts) were considered, the hierarchical cluster of array results was different (Figure 3B). Although the wild shrimp (West and East) were still clustered together and distinct from the domesticated groups, the domesticated shrimp could be further divided to 3 subgroups; 18-month-old shrimp alone, 14-month-old containing a single individuals of 18-month-old and 10-month-old containing a single individual of 14-month-old shrimp. No correlation according to gonadosomatic index (GSI) and ratio of sperm sac to testis weights (SS/TT) was observed.

**Table 1:** Experimental design for expression analysis between different *P. monodon *male broodstock types.

Experiment	Cy3	Cy5
***Gene expression levels in wild-caught broodstock***		
**I**	Juveniles (pooled, *n *= 114)	West-coast broodstock (individual, *n *= 3; GSI* = 1.3, 1.0, 0.7%)
		
**II**	Juveniles (pooled, *n *= 114)	East-coast broodstock (individual, *n *= 3; GSI = 0.9, 0.7, 0.5%)
		
***Gene expression levels in domesticated broodstock ***		
**III**	Juveniles (pooled, *n *= 114)	18-months-old domesticated broodstock (individual, *n *= 4; GSI = 0.8, 0.6, 0.6, 0.4%)
		
**IV**	Juveniles (pooled, *n *= 114)	14-months-old domesticated broodstock (individual, *n *= 4; GSI = 1.0, 0.8, 0.6, 0.4)
		
**V**	Juveniles (pooled, *n *= 114)	10-months-old domesticated broodstock (individual, *n *= 4; GSI = 1.0, 0.7, 0.6, 0.4)

*GSI is gonadosomatic index calculate as a percentage of testis weight by total body weight

**Figure 4:**
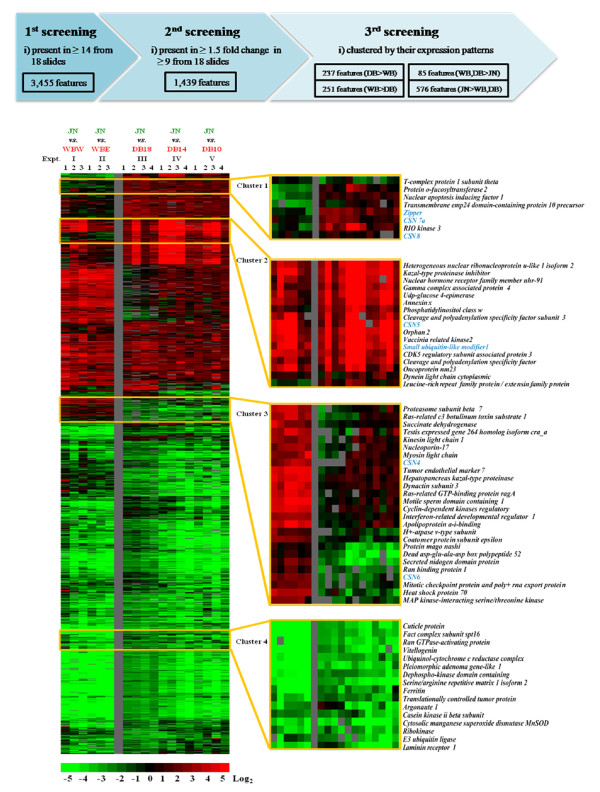
**Gene expression analysis by the UniShrimpChip comparing *P. monodon *transcript levels in testis of wild broodstock from the West and East coasts of Thailand (WBW and WBE, respectively), domesticated broodstock at 18, 14, and 10 months (DB-18, DB-14, and DB-10, respectively) using testes pooled from 114 juveniles (JN) as a common reference**. Testis from broodstock was labeled with Cy5 fluorescent dye (red) and pooled testes from juveniles with Cy3 fluorescent dye (green). Hierarchical clustering analysis of the 1,439 transcripts present in at least 14 of the 18 microarrays and whose expression differences were greater than 1.5 fold in at least 9 of the 18 microarrays. Clusters 1-4 show examples of various patterns of differentially expressed transcripts. Transcripts in blue letters were further characterized by quantitative real-time PCR.

From all 5,568 features of UniShrimpChip, 3,455 transcripts were selected for further analysis as they were present in at least 14 of the 18 microarrays (~80% of all the arrays). Of these, 1,439 transcripts exhibiting expression changes > 1.5 fold in at least 9 of the 18 microarrays (50% of all the arrays) were clustered according to their expression patterns (Figure 4). These differentially expressed transcripts were from different EST library origins. The majority of these genes were from testis (30.4%) and heart (28.9%), whereas the remaining transcripts were from ovary (14.8%), gill (10.7%), hemocyte (9.8%), hepatopancreas (2.6%), intestine (1.0%), and others (1.7%) (Additional file 1: Supplemental Figure S1).

From the 1,439 differentially expressed transcripts, 237 and 251 genes were expressed higher and lower in the domesticated than the wild shrimp, respectively. Comparing the expression levels between juveniles and broodstock, the microarray results revealed 576 genes with higher expression levels in juvenile, whereas only 85 genes were expressed higher in broodstock. Hierarchical cluster analysis of these differentially expressed transcripts revealed different gene groups (Figure 4). Examples of selected gene groups with different expression patterns were further examined (Figure 4).

Besides the comparison between the wild and domesticated gene expression profiles, the differences in gene expression levels among different ages within the domesticated groups (10-, 14-, and 18-month-old brooders compared with juveniles, experiments III-V) were also observed, albeit not as distinct. From the overall expression patterns, the 18-month-old profiles seemed different from the 10- and 14-month-old groups. For instance, the expression levels of the 18-month-old domesticated samples in Cluster 3 were slightly lower than the remaining samples from the domesticated group (Figure 4). Interestingly, from the different gene expression groups, a gene family of *constitutive photomorphogenic 9 signalosome (CSN) *homologs was presented in many clusters (Clusters I-III; Figure 4). The differential expression patterns of these gene subunits between the domesticated and wild groups raise a question whether they are involved in reproductive maturation in *P. monodon*.

### Gene Expression Levels Confirmed by Reverse-transcriptase Quantitative PCR (RT-qPCR)

**Figure 5:**
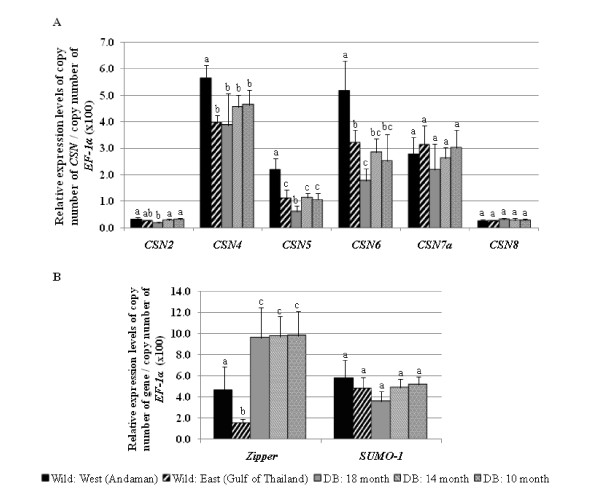
**Reverse-transcriptase quantitative PCR results of (A) *CSN *subunits, (B) *zipper *and *SUMO-1***. Black and patterned black bars are relative expression levels of wild broodstock from the West and East coasts of Thailand, respectively. Grey, lined grey and dotted grey bars are relative expression levels of domesticated broodstock (DB) at 18-, 14-, 10-month-old, respectively. Different letters above each bar designate significant differences in expression level (P < 0.05).

Expression levels of *CSN2, 4, 5, 6*, *7a*, and *8*, *small ubiquitin-like modifier 1(SUMO-1)*, and *zipper *homologues in testes of wild broodstock (the Andaman Sea, West and the Gulf of Thailand, East) and domesticated broodstock (18, 14, and 10 months old) were confirmed using reverse-transcriptase quantitative PCR (RT-qPCR, Figure 5) analysis.

Five *CSN *subunits (*CSN4, 5, 6, 7a and 8*) were present in the UniShrimpChip and their gene expression levels exhibited differential patterns in the microarray results. An additional subunit of this gene family, *CSN *subunit (*CSN2*), was just found from the recently sequenced testis cDNA library of *P. monodon *[10]; thus, the expression profiles of this subunit were included with the five subunits for RT-qPCR analysis.

The expression levels of *CSN4-6 *in the wild brooders obtained from the RT-qPCR agreed with the microarray data. The most distinct expression levels of the testes from the West coast of Thailand were significantly higher than the rest (*P *< 0.05). With the domesticated group of different ages, the expression levels of the *CSN2, CSN4, CSN5, CSN6 *and *CSN7a *were lowest in the samples of 18-month-old domesticated group; the lower expression levels of the 18-month-old domesticated group were significant for the *CSN2 *and *CSN5 *transcripts (P < 0.05). Also, agreed with the microarray results, the RT-qPCR results showed that the expression levels of *CSN7a *and *CSN8 *exhibited no significant difference among the samples (P > 0.05). Moreover, among the six subunits of the *CSN *genes studied in the work, *CSN2 *and *CSN8 *were expressed at much lower levels than other subunits (Figure 5A).

Besides the *CSN *gene family, two additional genes (*zipper *and *small ubiquitin-like modifier 1, SUMO-1)*, representing different expression patterns were also validated by the RT-qPCR analysis. From the microarray result, the *zipper *transcript was expressed higher in the domesticated group than in the wild samples (Figure 4; Cluster 1). The RT-qPCR results also revealed significantly higher expression levels of this transcript in the domesticated group than in the wild brooders (P < 0.05; Figure 5B). For *SUMO-1*, the microarray data suggested comparable expression levels in all groups (Figure 4, Cluster 2) which were confirmed by the RT-qPCR results that the expression levels were not significantly different (P > 0.05) in all samples tested (Figure 5B).

### Full-length cDNA Sequences and Phylogenetic Analysis of CSN5 and CSN6

**Figure 6:**
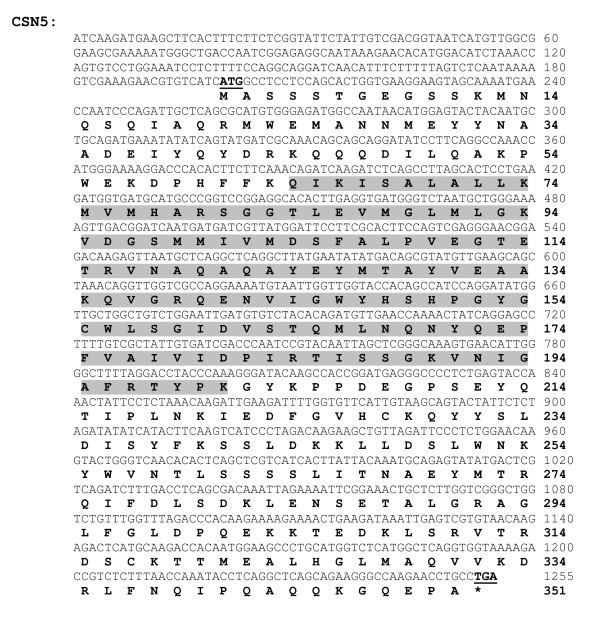
**The full-length cDNA sequence and deduced protein sequences of *CSN5 *(1,255 bp; ORF 1,056 bp, 351 aa) of *P. monodon***. Start (ATG) and stop (TAA) codons are illustrated in boldface and underlined. The deduced CSN5 protein contained a JAB/MPN (positions 64-201, *E-*value = 2.85e-50; highlighted).

**Figure 7:**
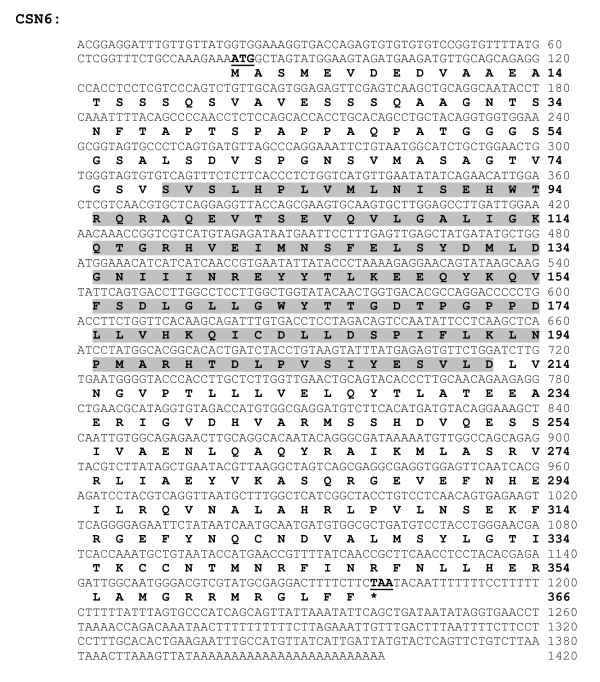
**The full-length cDNA sequence and deduced protein sequences of *CSN6 *(1,420 bp; ORF 1,101 bp, 366 aa) of *P. monodon***. Start (ATG) and stop (TAA) codons are illustrated in boldface and underlined. The deduced CSN6 protein contained a JAB/MPN (positions 78-212, *E-*value = 1.68e-15; highlighted).

The full-length cDNA sequence of the CSN gene family has never been reported in any crustacean. In this study, the full-length ORF of *PmCSN*5 and *PmCSN*6 (accession numbers are HQ416699 and HQ416700, respectively) were successfully isolated by RACE-PCR. For the *PmCSN*5, an ORF of 1,056 bp corresponding to 351 amino acids, with 5'UTR and 3'UTR of 199 and 459 bp (excluding the poly A tail), respectively, were identified (Figure 6). The predicted p*I *and molecular weight (MW) of the deduced CSN5 protein were 5.94 and 39.74 kDa, respectively. The closest similarity of this transcript was *CSN*5 of *Apis mellifera *(*E*-value = 1e-149). For the *PmCSN*6, the full-length cDNA sequence contains an ORF of 1,101 bp corresponding to 366 amino acids with 5'UTR and 3'UTR of 80 and 239 bp (excluding the poly A tail), respectively (Figure 7). The predicted p*I *and MW of the deduced PmCSN6 were 5.32 and 40.27 kDa, respectively. The closest similarity of the *PmCSN*6 transcript was *CSN6 *of *Xenopus laevis *(*E*-value = 1e-107). Both of the deduced PmCSN5 and PmCSN6 proteins contained a JAB/MPN domain (positions 64-201 with *E*-value = 2.85e-50 and positions 78-212, *E*-value = 1.68e-15, respectively; Figure 6 and 7).

**Figure 8:**
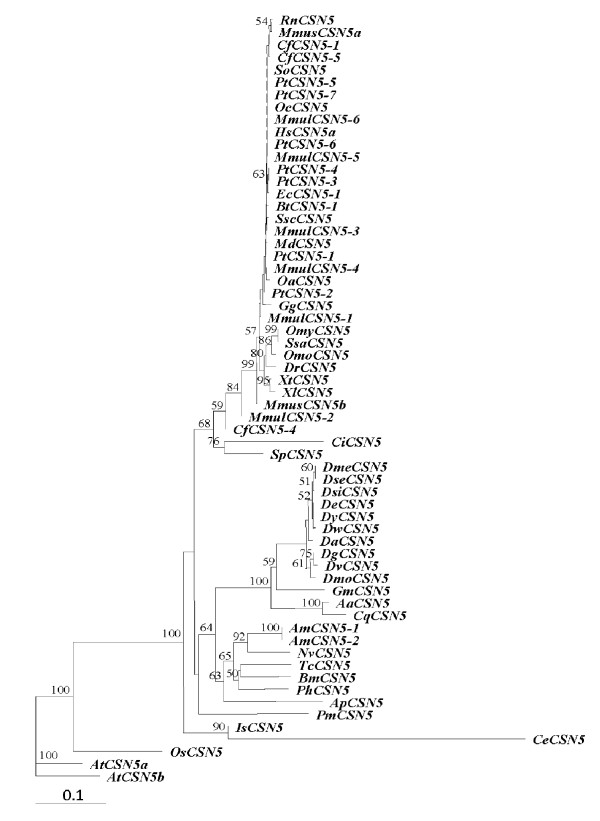
**A bootstrapped neighbor-joining tree illustrating relationships between CSN5 of various taxa**. Amino acid sequences of different isoforms of CSN5 from *Canis familiaris (CfCSN5-1*, XP_535093; *CfCSN5-3*, XP_859089; *CfCSN5-4*, XP_859121; *CfCSN5-5*, XP_859165), *Caenorhabditis elegans (CeCSN5*, P91001), *Danio rerio (DrCSN5*, NP_957019), *Xenopus (Silurana) tropicalis (XtCSN5*, NP_989109), *Xenopus laevis (XlCSN5*, NP_001086291), *Rattus norvegicus (RnCSN5*, NP_001020866), *Sus scrofa (SscCSN5*, NP_001098770), Salmo salar (*SsaCSN5*, ACH70627), *Oncorhynchus mykiss (OmCSN5*, NP_001158520), *Acyrthosiphon pisum (ApCSN5*, NP_001156462), *Gallus gallus (GgCSN5*, NP_001034400), *Osmerus mordax (OmCSN5*, ACO09632), *Oryctolagus cuniculus (OcCSN5*, XP_002710500), *Homo sapiens (HsCSN5a*, EAW86931), *Mus musculus (MmusCSN5a*, EDL14298; *MmusCSN5b*, EDL14299), *Culex quinquefasciatus (CqCSN5*, XP_001862398), *Ciona intestinalis (CiCSN5*, XP_002129245), *Bos Taurus (BtCSN5-1*, XP_583747), *Equus caballus (EcCSN5*, XP_001494265), *Ornithorhynchus anatinus (OaCSN5*, XP_001512862), *Monodelphis domestica (MdCSN5*, XP_001368118), *Strongylocentrotus purpuratus (SpCSN5*, XP_001190460), *Pan troglodytes (PtCSN5-1*, XP_001148926; *PtCSN5-2*, XP_001148995; *PtCSN5-3*, XP_001149144; *PtCSN5-4*, XP_522159; *PtCSN5-5*, XP_001162598; *PtCSN5-6*, XP_001162682; *PtCSN5-7*, XP_001162723), *Apis mellifera (AmCSN5-1*, XP_623806; *AmCSN5-2*, XP_623836), *Macaca mulatta (MmulCSN5-1*, XP_001097450; *MmulCSN5-2*, XP_001097549; *MmulCSN5-3*, XP_001097650; *MmulCSN5-4*, XP_001097759; *MmulCSN5-5*, XP_001097856; *MmulCSN5-6*, XP_001098042), *Arabidopsis thaliana (AtCSN5a*, Q9FVU9; *AtCSN5b*, Q8LAZ7), *Bombyx mori *(*BmCSN5*, NP_001138802), *Tribolium castaneum *(*TcCSN5*, XP_972769), *Nasonia vitripennis *(*NvCSN5*, XP_001599567), *Pediculus humanus corporis *(*PhCSN5*, XP_002422812), *Aedes aegypti *(*AaCSN5*, XP_001649479), *Glossina morsitans morsitans *(*GmCSN5*, ADD19140), *Drosophila melanogaster (DmeCSN5*, Q9XZ58), *Drosophila mojavensis *(*DmoCSN5*, XP_001998673), *Drosophila grimshawi *(*DgCSN5*, XP_001990340), *Drosophila virilis *(*DvCSN5*, XP_002056387), *Drosophila simulans *(*DsCSN5*, XP_002103175), *Drosophila ananassae *(*DaCSN5*, XP_001954897) *Drosophila yakuba *(*DyCSN5*, XP_002097599), *Drosophila erecta *(*DeCSN5*, XP_001980192), *Drosophila willistoni *(*DwCSN5*, XP_002074130), *Drosophila sechellia *(*DsCSN5*, EDW44858), *Sumatran orangutan *(*SoCSN5*, XP_002819198), *Ixodes scapularis *(*IsCSN5*, XP_002408598)and *Oryza sativa *Japonica Group(*OsCSN5*, NP_001054112) were retrieved from the GenBank and compared with that of *Penaeus monodon *(*PmCSN5*).

**Figure 9:**
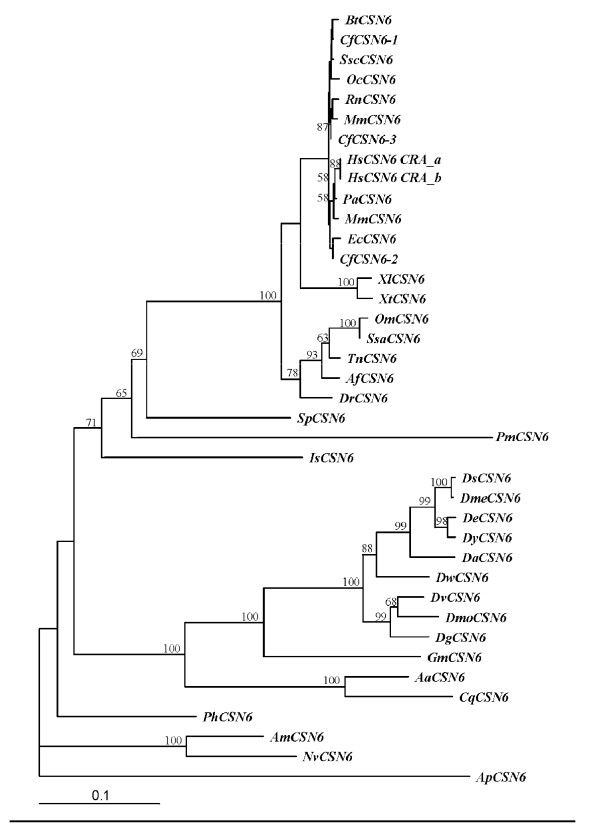
**A bootstrapped neighbor-joining tree illustrating relationships between CSN6 of various taxa**. Amino acid sequences of different isoforms of CSN6 from *Mus musculus *(*MmusCSN6*, O88545), *Xenopus (Silurana) tropicalis *(*XtCSN6*, Q07G98), *Oncorhynchus mykiss *(*OmCSN6*, NP_001154117), *Anoplopoma fimbria (AfCSN6*, ACQ58159), *Glossina morsitans morsitans (GmCSN6*, ADD19564), *Ixodes scapularis (IsCSN6*, XP_002401875), *Bos Taurus (BtCSN6*, NP_001095433), *Pongo abelii (PaCSN6*, NP_001124758), *Canis familiaris (CfCSN6-1*, XP_536866; *CfCSN6-2*, XP_860259; *CfCSN6-3*, XP_860298), *Culex quinquefasciatus (CqCSN6*, XP_001846513), *Equus caballus (EcCSN6*, XP_001505096), *Apis mellifera (AmCSN6*, XP_393678), *Macaca mulatta (MmulCSN6*, XP_001100125), *Sus scrofa (SscCSN6*, NP_001098769), *Homo sapiens (HsCSN6a*, EAW76601; *HsCSN6b*, EAW76602), *Xenopus laevis *(*XlCSN6*, NP_001084619), *Danio rerio *(*DrCSN6*, NP_001017768), *Salmo salar *(*SsaCSN6*, ACM09363), *Oryctolagus cuniculus *(*OcCSN6*, XP_002721904), *Rattus norvegicus *(*RnCSN6*, NP_001100599), *Nasonia vitripennis *(*NvCSN6*, XP_001606480), Aedes aegypti (*AaCSN6*, EAT33443), *Pediculus humanus corporis *(*PhCSN6*, XP_002423731), *Strongylocentrotus purpuratus *(*SpCSN6*, XP_001192086), *Tetraodon nigroviridis *(*TnCSN6*, CAG08305), *Acyrthosiphon pisum *(*ApCSN6*, NP_001155680), *Drosophila melanogaster *(*DmeCSN6*, AAF56022), *Drosophila ananassae *(*DaCSN6*, XP_001955632), *Drosophila mojavensis *(*DmoCSN6*, XP_002000957), *Drosophila sechellia *(*DsCSN6*, XP_002032224), *Drosophila yakuba *(*DyCSN6*, XP_002098311), *Drosophila virilis *(*DvCSN6*, XP_002053971), *Drosophila erecta *(*DeCSN6*, XP_001982256), *Drosophila willistoni *(*DwCSN6*, XP_002069934) and *Drosophila grimshawi *(*DgCSN6*, XP_001993979) were retrieved from the GenBank and compared with that of *Penaeus monodon *(*PmCSN6*).

A bootstrapped neighbor-joining tree indicated that vertebrate *CSN5s *are extremely conserved across different taxa (Figure 8). The newly characterized *PmCSN5 *was phylogenetically recognized as a member of invertebrate *CSN5s*. Although it showed the closest similarity with *Apis mellifera CSN5s *based on sequence similarity comparison, they are distantly related among each other phylogenetically. In addition, both *PmCSN5s *and *AmCSN5s *are more differentiated from other invertebrate *CSN5s *than that of other species. Interestingly, *PmCSN6 *was phylogenetically allocated to a member of the vertebrate rather than the invertebrate *CSN6s *along with that of *Strongylocentrotus purpuratus *and *Ixodes scapularis *(Figure 9).

## Discussion

### Identification and Characterization of Genes Related to Testicular Development by Microarray Analysis

A cDNA microarray, UniShrimpChip, was constructed from a total of 5,568 features from different EST libraries to cover unigenes of the black tiger shrimp *Peneaus monodon*. The usefulness of the UniShrimpChip was demonstrated in identification of differentially expressed genes possibly involved in different testicular development degrees in wild and domesticated male *P. monodon *brooders. The gene expression profiles of the wild brooders from West and East coasts of Thailand and domesticated brooders with different ages (10-, 14-, and 18-month-old) were compared to a same reference of testes pooled from 114 juvenile shrimp.

With every gene on the array, their overall gene expression patterns indicated the wild brooders, especially if they were from the same coast, exhibited similar patterns, whereas the domesticated groups expressed distinctively from the wild groups. Although actual differences between captive and wild male brooders are not well understood and some suggested no difference in sperm quality between the two [14], other early reports indicated that wild-caught brooders have higher reproductive success than in the captive counterparts [15,16]. Another study reported that using wild instead of captive males with either wild or domesticated females has successfully resolved a problem from low quality of offspring (B. Withyachumnarnkul, personal communication). Moreover, captive male *P. monodon *yields lower fertilization rates of zygotes and lower survival rates of offspring than wild male *P. monodon *[17]. The differences in the overall gene expression patterns between the wild and captive male brooders provide additional evidence to support that the difference in reproductive performance of the wild and captive broodstock might even be rooted at the transcriptomic level.

Considering the hierarchical clustering of differentially expressed genes, many interesting patterns were identified. For instance, the higher expression levels in domesticated broodstock in Clusters I and II might include genes whose functions are relevant to inhibiting maturation; therefore, their high expression levels might jeopardize the maturation levels in the captive male. Consequently, these genes should be further examined as they might be potential markers pinpointing maturity levels in this animal.

Various origins of the differential expressed genes from the microarray analysis confirm the versatility of UniShrimpChip. Although it is not surprising many (~30%) of the differentially expressed transcripts came from testis library, ~70% of the differentially expressed transcripts came from the other libraries combined. The significant contributions from other tissue libraries emphasize the benefit of including genes from various organs on the arrays to provide additional coverage in gene expression study.

### Validation of Gene Expression Profiles by Reverse-transcriptase Quantitative PCR (RT- qPCR)

To validate gene expression profiles of some selected transcripts belonging to different gene groups in microarray data, reverse-transcriptase quantitative PCR (RT-qPCR) was performed for *zipper*, *SUMO-1*, and six *CSN *genes. The RT-qPCR results mostly agreed with microarray results.

The expression levels of *SUMO-1 *in different brooder groups were validated by RT-qPCR, and its relatively stable expression levels from the RT-qPCR were in accord with the microarray result. *SUMO-1 *was first reported in *P. monodon *from the testis EST analysis and its transcript levels were found to be significantly lower in the domesticated than in the wild male brooders [9]. Similarly, we found the similar trend between the wild (West) and the domesticated samples, albeit not significantly different. The discrepancy between the previous and our reports could be explained by different of samples used in each study; for instance, the age of domesticated samples was not reported in the previous study.

For z*ipper*, its higher transcript levels in domesticated than in the wild group from the microarray data were confirmed by the RT-qPCR. In *Drosophila, zipper *encodes for a conventional non-muscle myosin heavy chain, NMMHC [18,19] whose functions involved in embryonic development [20]. The homologues of *zipper *are expressed in many species such as in chickens [21,22] and in humans [23],. Recently, Simons et al. reported that the two NMMHC-encoding genes were expressed differently in some rat tissues such as in brain and testis [23]. Though an actual function in testis of this gene family has not been established, the significant differences in expression levels between the wild and domesticated groups in our study might suggest that this gene could serve as a candidate biomarker indicating different maturation levels in testicular development in *P. monodon *brooders. Further characterization of this gene should be pursued to understand its molecular mechanism in testicular maturation and examine its roles on lower degree of male reproduction in captive brooders.

Another group of genes that were investigated through the RT-qPCR analysis was the *CSN *gene family. In addition to the five subunits (*CSN4, 5, 6, 7a, 8*) included in UniShrimpChip, *CSN2 *found from the testis EST library was also examined by the RT-qPCR. Compared to their expression patterns in the UniShrimpChip, the expression patterns of the five *CSN *genes were confirmed by the RT-qPCR results. The higher expression patterns of the *CSN4-6 *in the wild, especially from the West coast, than in the domesticated groups could suggest their relevance to the better testicular development of the West wild brooders. If these genes are involved in boosting testicular development, the significantly decreased expression levels of the *CSN5 *and *CSN6 *transcripts in the 18-month-old domesticated brooders from both microarray and RT-qPCR results would imply that the optimal mating age should be before 18 months. This information can be very useful for the *P. monodon *farming practice because an optimal age for reproductive maturity of a male brooder is not determined. In another related shrimp species *Litopenaeus vannamei*, a male brooder at 12 months is considered to give highest sperm quality based on spermatophore weight, sperm count, and percentage of normal sperm [24]. Therefore, the decrease in expressions of potential maturation-related genes at 18 months would be an undesired condition.

The COP9 signalosome (CSN) was first found in *Arabidopsis thaliana *mutants that exhibited constitutive photomorphogenesis, and hence its initial name as *constitutive photomorphogenesis 9 (COP9) *[25]. There are eight subunits of CSN found so far but not all eight subunits were found in all organisms [26]. In *P. monodon*, only six *CSN *subunits (*CSN 2, 4, 5, 6, 7, 8*) were found in the testis, ovary, hemocyte, and gill EST libraries[10,13]. The homologs of *CSN1 *and *CSN3 *have not been found in *P. monodon*.

Due to different names of COP9 signalosome homologues in various organisms, the names have been unified to CSN [27], whose system has been adopted in our study. Although the functions of the CSN are not well understood, there are many reports linking CSN with 19S proteasome based on their evolutionary relationship sharing the same number of eight subunits [28]. Some studies reported that E3 ubiquitin ligases were found to interact with the CSN, suggesting that CSN may function in assembling SCF (Skp1-cullin-F-box protein) complexes by bridging E3s and the proteasome [29-31]. However, clear biochemical and functional evidence of these hypotheses must be further verified.

From other previous studies in various organisms, loss of certain individual CSN subunits resulted in distinct phenotypes, suggesting that individual CSN subunits may have different and independent roles. For instance, in *Drosophila*, CSN has been implicated in oogenesis, embryogenesis, and neuronal development [32-34]. In rat testis, *CSN1 *was expressed differently at different developmental stages [35]. These links between some of these *CSN *subunits and testicular development in other organisms raise a question whether these genes are also involved in the male reproductive process of the black tiger shrimp.

### The Full-length cDNA Sequences of the CSN5 and CSN6

Among the *CSN *genes, *CSN5 *and *CSN6 *were expressed significantly lower in the 18-month-old domesticated group than any other groups but higher in the wild brooders from the West coast. Therefore, their full-length cDNA sequences were further characterized by RACE-PCR. For the first time in crustacean, the full-length *CSN*5 and *CSN*6 cDNA sequences were successfully characterized and revealed a JAB/MPN domain in both deduced proteins. This domain is one of the signature domains of a proteasome which was first observed at the N-terminus of the yeast proteins Mpr1p and Pad1p [36]. Among the eight subunits of the CSN, two subunits (CSN5 and CSN6) contain the JAB/MPN domain, whereas the remaining subunits contain a PCI domain [37]. The CSN5 homolog, JAB1, has been found in mouse and its RNA expression in testes was noticeably abundant in mouse embryonic tissue [38]. In *Drosophila*, the disruption of *CSN5 *gene resulted in lethality at the late larval or pupal stage, suggesting its essentiality for the development to the adult stage [34]. While *CSN5 *homologs in various organisms have been linked to reproduction and development, there are less numbers of reports linking *CSN6 *to reproduction. Instead, CSN6 was linked to apoptosis in vertebrates. When a cytoplasmic protein, Nod1, is activated as a part of apoptotic pathway to provide immune response against invading pathogens in mammals, CSN6, was cleaved from the COP9 complex, jeopardizing the complex's functions [39].

Phylogenetic analysis suggests that *CSN5 *is evolutionarily conserved in vertebrates and most invertebrates. Nevertheless, *PmCSN5 *was more differentiated from that of the other members of the Insecta (bootstrapping value of 63%). The molecular phylogeny reflects distant relationships between crustaceans and insect which is in agreement with that based on the conventional systematics. In contrast, *P. monodon*, *Strongylocentrotus purpuratus *and *Ixodes scapularis CSN6s *were allocated to be evolutionarily closer to the vertebrate than the invertebrate *CSN6s *(bootstrapping values of 65, 69 and 71%, respectively). This should not be the artifact from the phylogenetic analysis but the data implied that *CSN6 *in these and other invertebrates may be encoded by different loci. Southern blotting should be applied to determine whether *PmCSN6 *is encoded from more than one locus. However, this is beyond the scope of our present study.

## Conclusions

In summary, UniShrimpChip, a cDNA microarray containing non-redundant sequences of the *P. monodon *unigenes, was successfully constructed and employed in a high-throughput comparative gene expression analysis to identify genes potentially involved in the reproductive maturation differences between male brooders from the wild and domestication. Through comparison between different groups of male brooders (wild from the West and East coasts of Thailand and domesticated at 10, 14, 18 months), several genes were identified to exhibit differential expression patterns. Among those genes, the gene expression levels of *SUMO-1, zipper*, and six *CSN *genes were further validated by RT-qPCR and the results mostly agreed with the microarray data. Because of their significant decreased transcript levels in the 18-month-old captive brooders, the full-length cDNA sequences of the two *CSN *genes, *CSN5 *and *CSN6*, were further characterized to compare with other organisms.

This study demonstrates the usefulness of the UniShrimpChip for comparative gene expression study. For the first time, the findings reveal the possibility of the *CSN *gene homologs to be involved in testicular maturation and development in the black tiger shrimp. Though further studies to pinpoint the molecular mechanisms of these genes in testicular development are required, through microarray technique, these results open a new door to narrow down candidate genes relevant to an important developmental process of this important animal.

## Methods

### Unigene Assembly and EST Clone Selection

Unigenes and contigs of previously sequenced ESTs from the *P. monodon *EST database (PmDB: http://pmonodon.biotec.or.th) were analyzed using ESTplus, an integrative system for comprehensive and customized EST analysis and proteomic data matching [40,41]. An e-value <1e-05 was taken to indicate a significant probability of homology for both nucleotides and amino acids. The sequences were grouped in different gene ontology categories according to [42] using a universal tool for annotation, visualization and analysis, Blast2GO [43]. These unigenes and contigs were identified for the synthesis of array probes. If the plasmids containing these sequences were available from the EST libraries, the plasmids were used as templates for probe amplification. Otherwise, gene-specific primers were designed for amplification from the first strand cDNA using Primer3 program [44]. Primer sequences are available on requested.

### Microarray Probe Synthesis and Fabrication

In total, 5,568 sequences were amplified by PCR method, which was carried out in a 96-well PCR plate (Axygen) using a Veriti 96 well Thermocycler (ABI). For the plasmid template, a reaction mixture contained 0.3 mM dNTPs, 1× PCR buffer, 2 mM MgCl2, 0.4 μM each of LM13-F (5'-CACGACGTTGTAAAACGACGGCCAG-3') and LM13R (5'-CATGGTCATAGCTGTTTCCTGT-3'), and 1.25 U of *i-Taq*™ DNA Polymerase (iNtRON Biotechnology, Inc; Korea). The PCR reactions were carried out for 35 cycles at the following settings: 94°C for 30 s, 60°C for 25 s, and 72°C for 3 min. For the first strand cDNA template, The PCR reactions contained 0.4 mM dNTPs, 1× PCR buffer, 0.6 μM each gene-specific primer. Amplification was carried out for 38 cycles at the following settings: 94°C for 30 s, 57°C for 60 s, and 72°C for 3 min. Five microliters of the amplicons were electrophoretically analyzed in a 1% agarose gel prepared in 0.5× TBE buffer. The PCR products were purified using MultiScreen^® ^PCR 96 (Millipore) filtration plate method, air-dried and resuspended with 7 μl 3× SSC (Invitrogen) giving the approximate concentration of 250-500 ng/μl. The plates were covered tightly and left at 4°C overnight. The resuspended products were transferred from 96-well to 384-well print plates using a liquid handler Multi Probe II Plus Ex (PerkinElmer). The cDNA microarrays were fabricated using Nanoprint LM210 microarray spotter (ArrayIt, USA) equipped with 24 pins (946MP3) in a 4 × 6 arrangement on GAPS II amino-silane coated slides (CORNING). Each amplicon was spotted in duplicate. The printed slides were UV-crosslinked at 200 mJ of energy (GS Gene Linker UV chamber, BIO-RAD) and stored at room temperature in a dry incubator.

### Post-processing, Labeling, Hybridization and Analysis

The slides were post-processed to block unused surfaced using a BSA-based method as previously described [12]. DNase-treated RNA samples were converted to the first-strand cDNAs and labeled with aminoallyl-dUTP (aa-dUTP; Sigma) using a LabelStar Array kit (Qiagen). The aa-dUTP samples were cleaned up using a Microcon YM-30 filter (Millipore) and resuspended in 6 μl of 0.1 M sodium borate buffer (pH 8.7). The aa-dUTP cDNA was fluorescently labeled with Cy3- or Cy5- dyes (GE Healthcare) at room temperature for 1 h. The unincorporated dye was removed using a Microcon YM-30 filter and the purified probe was resuspended in 6 μl of TE. The Cy3- and Cy5- samples were mixed together in a hybridization buffer (Pronto! Microarray hybridization kit, CORNING) in 30 μL of reaction volume solution and denatured at 95°C in the dark for 3 min before applying onto the post-processed slide with a glass cover slip (22 × 32 mm, Deckglasser) for each experiment.

Hybridization was performed overnight (12-16 hr) in a sealed hybridization chamber (CORNING) at 42°C. The hybridized arrays were immersed into a pre-warmed washing buffer I (2× SSC and 0.1% SDS) to remove a cover slip before transferred to wash in the same buffer for 5 min with gently shaking. The arrays were washed in a washing buffer II (0.2× SSC) and a washing buffer III (0.05× SSC) for 5 min each at a room temperature with gently shaking. The arrays were then dried by centrifugation at 1,000 rpm for 5 min at room temperature (Allegra X-22 R, Beckman coulter).

The hybridized slides were scanned with GenePix 4000B (Molecular Devices, Sunnyvale, CA) and processed using GenePix Pro version 6.1. The data analysis process was described previously [12]. The microarray data have been deposited in NCBIs Gene Expression Omnibus with GEO accession number GSE21890 at http://www.ncbi.nlm.nih.gov/geo/.

### RNA Samples and Microarray Experiments

Total RNA was extracted from *P. monodon *organs using TRI-Reagent according to manufacturer's instruction (Molecular Research Center, USA). Contaminated genomic DNA was removed by treatment with DNase I (0.15 U/μg total RNA) at 37°C for 30 min. The quality and quantity of the RNA were assessed on conventional agarose based electrophoresis and NanoDrop (ND-8000) before labeling for microarray experiments.

For all microarray experiments in this study (Table 1), all Cy3 samples were from testis samples pooled from 114 individuals of 4-month-old juveniles.

For experiments I-II, Cy5 sample was testis from individual wild-caught male brooder from Andaman Sea (West, *n *= 3) and Gulf of Thailand (East, *n *= 3), respectively.

For experiments III-V, Cy5 sample was testis from individual domesticated brooder at different ages (18-, 14-, and 10-month-old, respectively; *n *= 4 for each age group) from the Broodstock Multiplication Center (BMC, Burapha Univeristy, Chanthaburi province, Thailand).

### Reverse-transcriptase Quantitative PCR (RT-qPCR)

Gene-specific primers with the expected product of 120-150 bp long were designed for each transcript (Table 2). For construction of the standard curve of each transcript, a plasmid containing the transcript was constructed by the cloning the PCR product of the transcript into a pGEM-T easy vector and transforming the resulting vector into *E. coli *JM109. The plasmid was extracted and used as the template for construction of the standard curve by 10-fold serial dilutions (10^3^-10^9 ^copy numbers). Reverse-transcriptase quantitative PCR (RT-qPCR) reactions were carried out in the strip tubes.

**Table 2:** Primer pairs for reverse-transcriptase quantitative PCR and RACE-PCR

Primer		Sequence	Size (bp)	PCR Efficiencies
**Reverse-transcriptase quantitative PCR**				
***CSN2***		F: 5'-GAGGATGACCTGAAGAAGGGAACA-3'	145	97.1%
		R: 5'-TAAGTGGATGGGGAATTGCCG-3'		
***CSN4***		F: 5'-GCAGGGCAGCAGCGTTCAAGA-3'	111	96.2%
		R: 5'-ACGGATGATGCGGTCCAGGTG-3'		
***CSN5***		F: 5'-CGAGGGAACGGAGACAAGAGT-3'	148	97.5%
		R: 5'-TCCAGACAGCCAGCAACCATA-3'		
***CSN6***		F: 5'-CAGTTTCTCTTCACCCTCTGGTCATA-3'	115	97.6%
		R: 5'-GTTTGTTTTCCAATCAAGGCTCC-3'		
***CSN7A***		F: 5'-CTCTTTGCTTATGGTGTTTATGCTGA-3'	128	99.8%
		R: 5'-ATACTCTTCTGCCCCGTTGCC-3'		
***CSN8***		F: 5'-TGGTTGCTCGGGCATATACATC-3'	128	99.0%
		R: 5'-AATCATTCTCGTGGGTTCGTCAT-3'		
***SUMO-1***		F: 5'-AGAAGGGGAAGGGAACGAATACA-3'	148	98.2%
		R: 5'-ACGCAGCGATGCTACAGGGA-3'		
***Zipper***		F: 5'-TGTGAGACAGACCTGAACGACCTT-3'	122	95.4%
		R: 5'-ATCTACACGCATCATTGCTTGGGC-3'		
***EF-1α***		F: 5'-TTCCGACTCCAAGAACGACC-3'	122	96.5%
		R: 5'-GAGCAGTGTGGCAATCAAGC-3'		
**RACE-PCR**				
***CSN5***	3' RACE-PCR	F: 5'-GACCTACCCAAAGGGATACAAGCCACCG-3'		
***CSN6***	5' RACE-PCR	R: 5'-TGGGCTAACATCACTGAGGGCACTACCG-3'		
	3' RACE-PCR	F: 5'-GTCAGCGAGGCGAGGTGGAGTTCAAT-3'		

Subsequently, RT-qPCR was used to analyzed expression levels of the transcripts in testes from juveniles (JN, 4-month-old, *n *= 6), 10-month-old domesticated broodstock (DB) with an average gonadosomatic index (GSI calculated from the percentage of gonad weight from body weight) of 0.69 ± 0.25 (*n *= 5), 14-month-old DB (average GSI = 0.51 ± 0.05; *n *= 5), 18-month-old DB (average GSI = 0.53 ± 0.21; *n *= 7), wild broodstock from Andaman Sea (West) and Gulf of Thailand (East) (average GSI = 1.14 ± 0.26 and 0.74 ± 0.12, respectively; *n *= 5 for each group) Each reaction was performed in a 20-μl total reaction volume containing 2× iQ™ SYBR^® ^Green Supermix (Bio-Rad), 200 ng of first strand cDNA template, and 0.2 μM of each primer. Cycling parameters were 95°C for 2.5 min; followed by 40 cycles of 95°C for 30 s, 58°C for 20 s, and 72°C for 30 s. The specificity of PCR products was confirmed by melting curve analysis performed from 55°C-95°C with a continuous fluorescent reading with every 0.5°C increment. The relative expression levels of copy number of the target gene to that of a housekeeping gene *EF-1α *in different sample groups were statistically tested by ANOVA followed by Duncan's new multiple range test or Tukey test (*P *< 0.05). Note that *EF-1α *have been widely used as a housekeeping gene for gene expression profile analysis in *P. monodon *[10,45-53].

### Rapid Amplification of cDNA Ends-Polymerase Chain Reaction (RACE-PCR) of CSN5 and CSN6

Gene-specific primers for RACE-PCR of *P. monodon CSN5 *and *CSN6 *were designed (Table 2). RACE-PCRs of these genes were carried out using a BD SMART RACE cDNA Amplification Kit following the protocol recommended by the manufacturer (BD Biosciences Clontech). The amplified fragment of each gene was electrophoretically analyzed, purified from an agarose gel before cloning into pGEM-T Easy vector (Promega) and sequenced (Sambrook and Russell, 2001). The full-length cDNA was assembled from the EST sequences and RACE-PCR products (hereafter called *PmCSN5 *and *PmCSN6*; accession numbers are HQ416699 and HQ416700, respectively). The functional domain of deduced amino acids of these genes was analyzed using SMART http://smart.embl-heidelberg.de/. The p*I *and molecular weight were estimated using ProtParam http://www.expasy.org/tools/protparam.html.

### Phylogenetic analysis of PmCSN5 and PmCSN6

The amino acid sequences of *P. monodon CSN5 *and *CSN6 *proteins were phylogenetically compared with those from other species found in GenBank http://ncbi.nlm.nih.gov. Multiple alignments were carried out with ClustalW [54]. Bootstrapped neighbor-joining trees [55] were constructed with the Seqboot, Prodist, Neighbor, and Consense routines in PHYLIP http://evolution.gs.washington.edu/phylip.html and appropriately illustrated with Treeview http://taxonomy.zoology.gla.ac.uk/rod/treeview.html.

## Authors' contributions

RL performed microarray experiments, gene expression analysis experiments, and full-length characterization. UU and NK constructed cDNA microarray chips. NK and SK conceived the work. RL, UU, SK and NK wrote the manuscript. All authors read and approved this manuscript.

## Supplementary Material

Additional file 1 **Distribution of differential expressed genes according to different tissues**. Pie graph shows distribution of differential expressed genes according to different tissues. Click here for file
