# Supplementary figures and images for: A cDNA microarray, UniShrimpChip, for identification of genes relevant to testicular development in the black tiger shrimp (Penaeus monodon)
Source: BMC Mol Biol. 2011 Apr 12;12:15. doi: 10.1186/1471-2199-12-15 (PMC3094230; doi:10.1186/1471-2199-12-15)

**
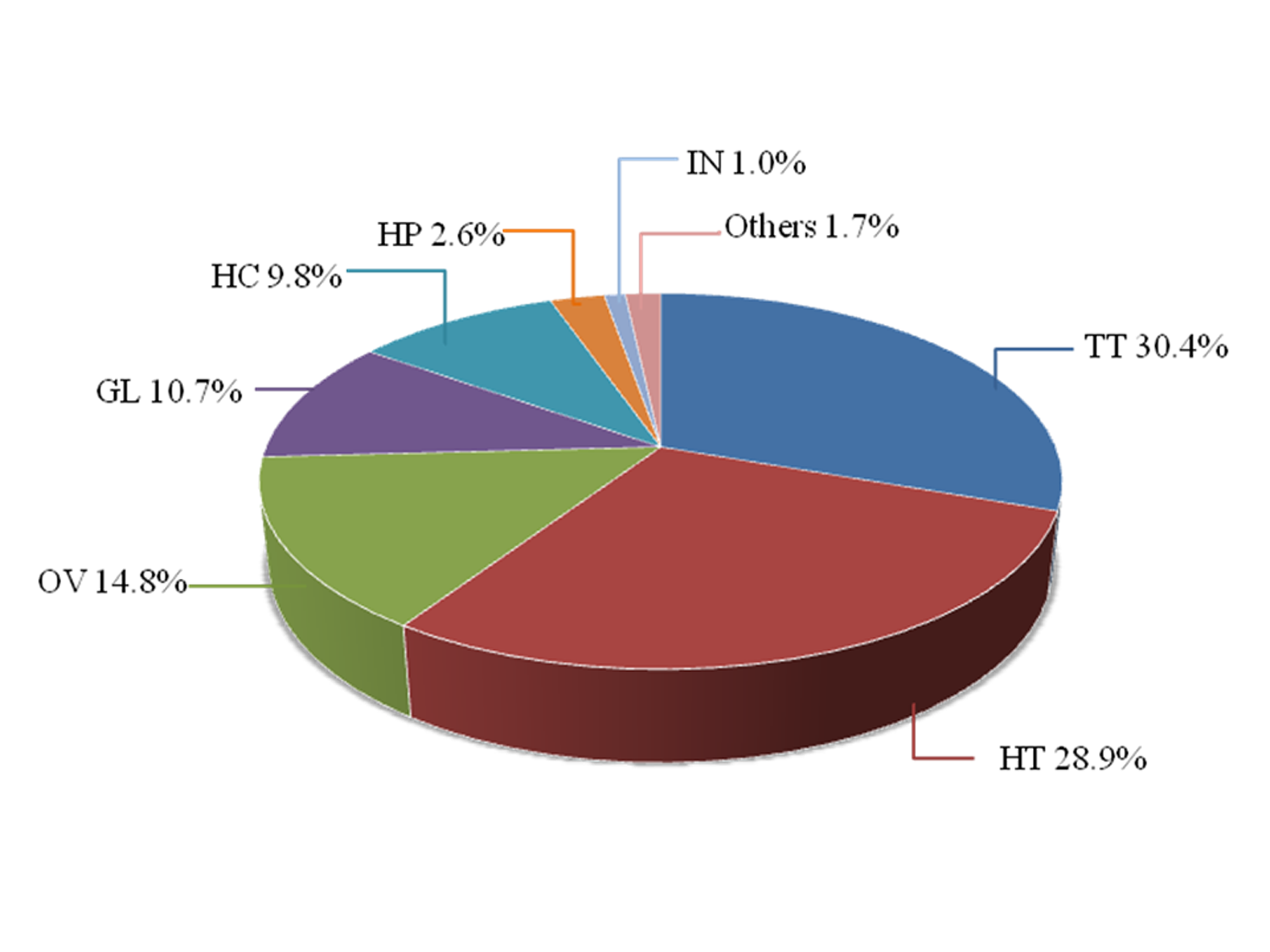
**

Supplement: Additional file 1 — Distribution of differential expressed genes according to different tissues. Pie graph shows distribution of differential expressed genes according to different tissues. [file 1471-2199-12-15-S1.DOC]
